# Validation of Potential Protein Markers Predicting Chemoradioresistance in Early Cervical Cancer by Immunohistochemistry

**DOI:** 10.3389/fonc.2021.665595

**Published:** 2021-07-19

**Authors:** Soo Young Jeong, Joon-Yong Chung, Sun-Ju Byeon, Chul Jung Kim, Yoo-Young Lee, Tae-Joong Kim, Jeong-Won Lee, Byoung-Gie Kim, Ye Lin Chae, So Young Oh, Chel Hun Choi

**Affiliations:** ^1^ Departments of Obstetrics and Gynecology, Samsung Medical Center, Sungkyunkwan University School of Medicine, Seoul, South Korea; ^2^ Laboratory of Pathology, Center for Cancer Research, National Cancer Institute, National Institutes of Health, Bethesda, MD, United States; ^3^ Departments of Pathology, Dongtan Sacred Heart Hospital, Hallym University College of Medicine, Hwaseong, South Korea; ^4^ Departments of Obstetrics and Gynecology, College of Medicine, Konyang University, Daejeon, South Korea

**Keywords:** HER2, CAIX, ERCC1, immunohistochemistry, chemoradioresistance, uterine cervical neoplasm

## Abstract

**Background:**

In a previous study, a proteomic panel consisting of BCL-2, HER2, CD133, CAIX, and ERCC1 significantly predicted survival in patients with locally advanced cervical cancer. However, the prognostic significance of these proteins has not been assessed in early cervical cancer. The present study investigated the clinical significance and chemoradioresistance prediction power of these proteins in patients with early-stage cervical cancer.

**Materials and Methods:**

BCL-2, HER2, CD133, CAIX, and ERCC1 expression was determined by the immunohistochemical staining of 336 cervical cancer tissue microarrays. The associations of these proteins with clinicopathologic characteristics and disease progression were assessed.

**Results:**

There was a trend of low CAIX expression (p=0.082) and high ERCC1 expression (p=0.059) in patients with a favorable response to adjuvant radiation. High HER2 expression was significantly associated with shorter disease-free survival (DFS) in the total group (5-year DFS of 80.1% *vs*. 92.2%, p=0.004). A prognostic significance remained in multivariate analysis (Hazard ratio, HR=2.10, p=0.029). In the adjuvant radiation group, low CAIX and high ERCC1 expression indicated significantly unfavorable DFS (75.0% *vs*. 89.0%, p=0.026 and 76.8% *vs*. 88.6%, p=0.022, respectively). Low CAIX expression remained an independent prognostic marker in multivariate analysis (HR=0.45, p=0.037). The combined molecular-clinical model using random survival forest method predicted DFS with improved power compared with that of the clinical variable model (C-index 0.77 *vs*. 0.71, p=0.006).

**Conclusion:**

HER2, CAIX, and ERCC1 expression can be predictive protein markers for clinical outcomes in early cervical cancer patients treated primarily with radical surgery with or without adjuvant radiation.

## Introduction

Cervical cancer is the fourth most common malignancy and the leading cause of cancer-related death in women in developing countries (1). In the early stage, radical hysterectomy and adjuvant radiation with or without chemotherapy is the primary treatment and performed if there are risk factors. Although the prognosis is generally good, once disease recurs, there are limited options. The choice of adjuvant treatment depends not only on the side effects but also on the efficacy of the treatment. Each patient has a varied response to adjuvant radiation and/or chemotherapy; therefore, the prediction of the response to each treatment is important.

Several clinical factors that can predict the response to radiotherapy in cervical cancer have been investigated. Larger tumor size, regional metastases, and histologic subtypes are associated with a poor response to radiation (2, 3). Also, high tumor vascularity (4) and tumor hypoxia (5) contribute to radiation therapy resistance and are related to poor survival rates. Hyperthermia during radiotherapy can also make cancer cells more sensitive and affect the outcome of radiotherapy (6). However, these factors alone cannot accurately predict chemoradiosensitivity. Therefore, new markers with molecular approaches using genes or proteins are needed to predict the clinical outcomes of cancer patients more accurately.

Kitahara et al. identified a set of 62 genes that might be of great benefit for diagnosing the radiosensitivity of individual cervical squamous cell carcinomas (7). The expression of several apoptotic regulators, such as the B cell lymphoma-2 (BCL-2) family of proteins (BCL-2, BCL-XL, and BAX) and p53, may correspond to cervical cancer cell radiosensitivity (8, 9). In addition, the expression level of cyclooxygenases (COXs) (10, 11), epidermal growth factor receptor (EGFR), and vascular endothelial growth factor (VEGF) (12, 13) have been associated with radiosensitivity in several experimental studies. Clinicians have based methods of treatment on clinical factors; however, it is necessary to develop and identify biomarkers in an era of personalized therapy.

Previously, we have identified protein markers predicting survival using reverse-phase protein array (RPPA) in locally advanced cervical cancer. We used 181 locally advanced cervical cancer tissues to assess the expression levels of 22 selected protein markers using well-based RPPAs. The expression signals in well-based RPPA were correlated with data from western blot and immunohistochemistry (IHC). We also found that a panel of proteins, BCL-2, HER2, CD133, CAIX, and ERCC1, can be predictors of overall survival in patients with locally advanced cervical cancer treated with concurrent chemoradiotherapy (CCRT) by calculating risk scores, which were the sum of estimated coefficients from age, cancer stage, and the protein panel (14).

In this study, because a correlation existed between the protein panel and cancer prognosis or chemoradiation resistance in locally advanced cervical cancer, we assessed if the protein panel could predict disease prognosis or treatment response in patients with early-stage cervical cancer who had been treated using radical hysterectomy with or without adjuvant radiation. We analyzed the prognostic significance of these markers using IHC in tissue microarrays.

## Materials and Methods

### Patients and Tumor Samples

In this study, we retrieved the data of a total of 336 early-stage cervical cancer patients who were treated in the Department of Gynecologic Oncology, Samsung Medical Center (Seoul, South Korea), Sungkyunkwan University School of Medicine between 2002 and 2009. Tissue samples and medical records were obtained from patients who had signed an informed consent form, which was approved by the Institutional Review Board of Samsung Medical Center (IRB no. SMC 2009-09-002 and 2015-07-122; Seoul, South of Korea).

For the primary treatment, all patients underwent radical hysterectomy with or without pelvic/para-aortic lymph node dissection. In addition, patients received adjuvant radiotherapy with or without CCRT if the following risk factors were found; larger tumor size (more than 4 cm), lymphovascular invasion, deep stromal invasion (more than half), positive resection margin, parametrial invasion, or pelvic/para-aortic lymph node metastasis. After primary treatment, all patients received adequate follow-up treatment. During this period, patients underwent physical examination, Pap smears, and tumor marker measurements every 3 months for the first 2 years, and every 6 months for the next 3 years. HPV test was performed in not all patients but in those decided by the clinicians. HPV typing was done by using HPV type-specific primers. Cancer staging was classified by international federation of gynecology and obstetrics (FIGO) staging 2008. Imaging studies, such as chest radiography and abdominopelvic and/or chest computed tomography (CT), were conducted every 3–6 months for the first 2 years and then 6–12 months for the next 3 years.

Disease-free survival (DFS) was defined as the time interval from treatment to the first evidence of recurrence or the last follow-up. To examine the association between protein expression and chemoradiotherapy resistance, we defined ‘resistant response’ as recurrence within 3 years from adjuvant therapy and ‘sensitive response’ as no recurrence over three years from adjuvant therapy (15).

### Tissue Microarray and Immunohistochemistry

Tissue microarrays (TMAs) were constructed from tissue blocks used for routine pathologic evaluation. In each case, areas with the most representative histology were selected, and three 0.6 mm cylindrical tissue cores were taken from formalin-fixed paraffin-embedded (FFPE) tissue blocks and extruded into the recipient paraffin block. To check the adequacy of tissue sampling, sections from each microarray were stained with hematoxylin and eosin and examined by light microscopy.

Immunohistochemical staining of BCL-2, HER2, CD133, CAIX, and ERCC1 was performed on 4 μm sections of TMAs and was performed using a standard streptavidin–peroxidase method as described previously (16). In order to prevent possible antigenicity loss during slide ageing (delay between cutting section and IHC staining), we used fresh-cut sections from original TMA blocks. After deparaffinization by using xylene and dehydration with graded ethanol, heat-induced antigen retrieval was performed for 20 minutes in an antigen retrieval buffer (Dako, Carpinteria, CA) of pH 6.0 (for BCL-2, HER2, CD133, and CAIX) and pH 8.0 (for ERCC1) in a pressure cooker (Pascal, Dako). Endogenous peroxidase activity was blocked with 3% H2O2 for 10 minutes at room temperature. The sections were incubated with primary antibodies. A detailed list of antibodies and adequate dilutions are provided in **Supplementary Table 1**. The primary antibodies were applied to test sections and positive-control sections for an adequate incubation time. Also, negative control slides were incubated by omitting the primary antibodies and no detectable staining was observed. The antigen–antibody reaction was detected with Dako EnVision+ Dual Link System-HRP (Dako) and DAB+ (3,3′-diaminobenzidine; Dako). Tissue sections were lightly counterstained with hematoxylin and then examined by light microscopy.

### Quantitative Evaluation of Immunostaining

The evaluation of immunohistochemical staining was scored independently by two investigators (SJB and CHC) without knowledge of the clinicopathological findings. The intensity of staining was categorized as 0, 1+, 2+, and 3+ according to the distribution pattern across cores. The overall protein expression was measured as the mean value of histoscores, which is a result of multiplying the intensity score (0–3) and the percentage of stained cells, with a maximum of 300. For the survival analysis, expression values were dichotomized (high *vs*. low) with the cut-off values showing the most discriminative power (histoscore of 1 for BCL-2, 1 for HER2, 1 for CD133, 6 for CAIX, and 50 for ERCC1).

### *In Silico* Analysis Using GSE44001

To examine the correlation between each protein expressions and corresponding mRNA expressions, data from the Gene Expression Omnibus (GEO) were analyzed as described previously (17). We downloaded the GES44001 dataset from the GEO website (https://www.ncbi.nlm.nih.gov/geo/query/acc.cgi?acc=GSE44001) and the samples of 300 patients were available. The analysis was carried out in the patients included in both studies.

### Statistical Analysis

We performed statistical analysis using R 3.3.2 (Vienna, Austria; http://www.R-project.org). The expression levels of the proteins according to the clinicopathological characteristics were analyzed using Student’s t-test or Mann–Whitney U-test. Analysis of the Spearman’s rho coefficient was used to assess the correlation between proteins and mRNA expression. Analyses for survival distributions were performed by the Kaplan–Meier method and comparison between survival and each parameter was done with the log-rank test. We used the Cox proportional hazards model to evaluate the prognostic predictors of DFS.

To identify the predictive power of integrating the molecular data with clinical variables, we modified the random survival forest (RSF) method to include both clinical and molecular data (18). We used clinical data (FIGO stage, lymph node metastasis, tumor histology, tumor size, and parametrial invasion) to build the clinical RSF model and combined the molecular-level features with the clinical variables to build a new RSF model. A concordance index (C-index), which is a nonparametric measure to quantify the discriminatory power of a predictive model, was calculated and compared between the clinical and combined models using the Wilcoxon signed-rank sum test (19). All p-values were two-sided, and we considered p-values of less than 0.05 as statistically significant.

## Results

### Clinicopathological Characteristics of Patients

The clinicopathological characteristics of 336 patients are summarized in **Table 1**. The mean age of the patients was 49 years and 45 patients (13.4%) with IB2 or IIB were included because they were primarily treated with radical surgery and adjuvant radiotherapy with or without chemotherapy. In total, 291 (86.6%) patients were stage IIA or less and 256 (76.2%) patients had squamous cell carcinoma (76.2%). In165 patients, a HPV infection test was performed and 128 (77.6%) had high-risk types of HPV. Lymph node metastasis was found in 80 (23.8%) patients, parametrial invasion in 31 (9.2%), and positive resection margin in 13 (3.9%). Overall, 165 patients (49.1%) were treated with adjuvant radiation after radical surgery.

**Table 1:** Clinicopathological characteristics of 336 early cervical cancer patients according to adjuvant treatment.

	Operation group (*n* = 171)	Operation and adjuvant treatment group (*n *= 165)	*p* value
Age	48 (41-58)	48 (42-56)	0.825
Stage			<0.001
IB1/IIA	161 (94.2%)	130 (78.8%)	
IB2/IIB	10 (5.8%)	35 (21.2%)	
Histology			0.647
SCC	128 (74.9%)	128 (77.6%)	
AD/ASC	43 (25.1%)	37 (22.4%)	
Tumor size			<0.001
≤4 cm	155 (90.6%)	101 (61.2%)	
>4 cm	16 (9.4%)	64 (38.8%)	
High risk HPV infection			0.706
Negative	18 (20.7%)	19 (24.4%)	
Positive	69 (79.3%)	59 (75.6%)	
LVSI			<0.001
Negative	128 (74.9%)	74 (44.8%)	
Positive	43 (25.1%)	91 (55.2%)	
Depth of invasion			<0.001
≤50%	93 (54.4%)	15 (9.1%)	
>50%	78 (45.6%)	150 (90.9%)	
Parametrial invasion			<0.001
Negative	167 (97.7%)	138 (83.6%)	
Positive	4 (2.3%)	27 (16.4%)	
Resection margin			0.020
Negative	169 (98.8%)	154 (93.3%)	
Positive	2 (1.2%)	11 (6.7%)	
Lymph node metastasis			<0.001
Negative	158 (92.4%)	98 (59.4%)	
Positive	13 (7.6%)	67 (40.6%)	

SCC, squamous cell carcinoma; AD, adenocarcinoma; ASC, adenosquamous cell carcinoma; LVSI, lymphvascular space invasion.

Of the patients treated with adjuvant radiation, 113 patients (85.0%) were classified as chemoradiosensitive and 20 patients (15.0%) were classified as chemoradioresistant. The clinicopathological characteristics of patients with adjuvant treatment are shown in **Supplementary Table 2**. In total, 76 patients (57.1%) had CCRT and 57 (42.9%) patients had radiotherapy. Squamous cell carcinoma was more sensitive to radiation than adenocarcinoma or adenosquamous carcinoma (p=0.001).

### BCL-2, HER2, CD133, CAIX, and ERCC1 Protein Expression

**Figure 1:**
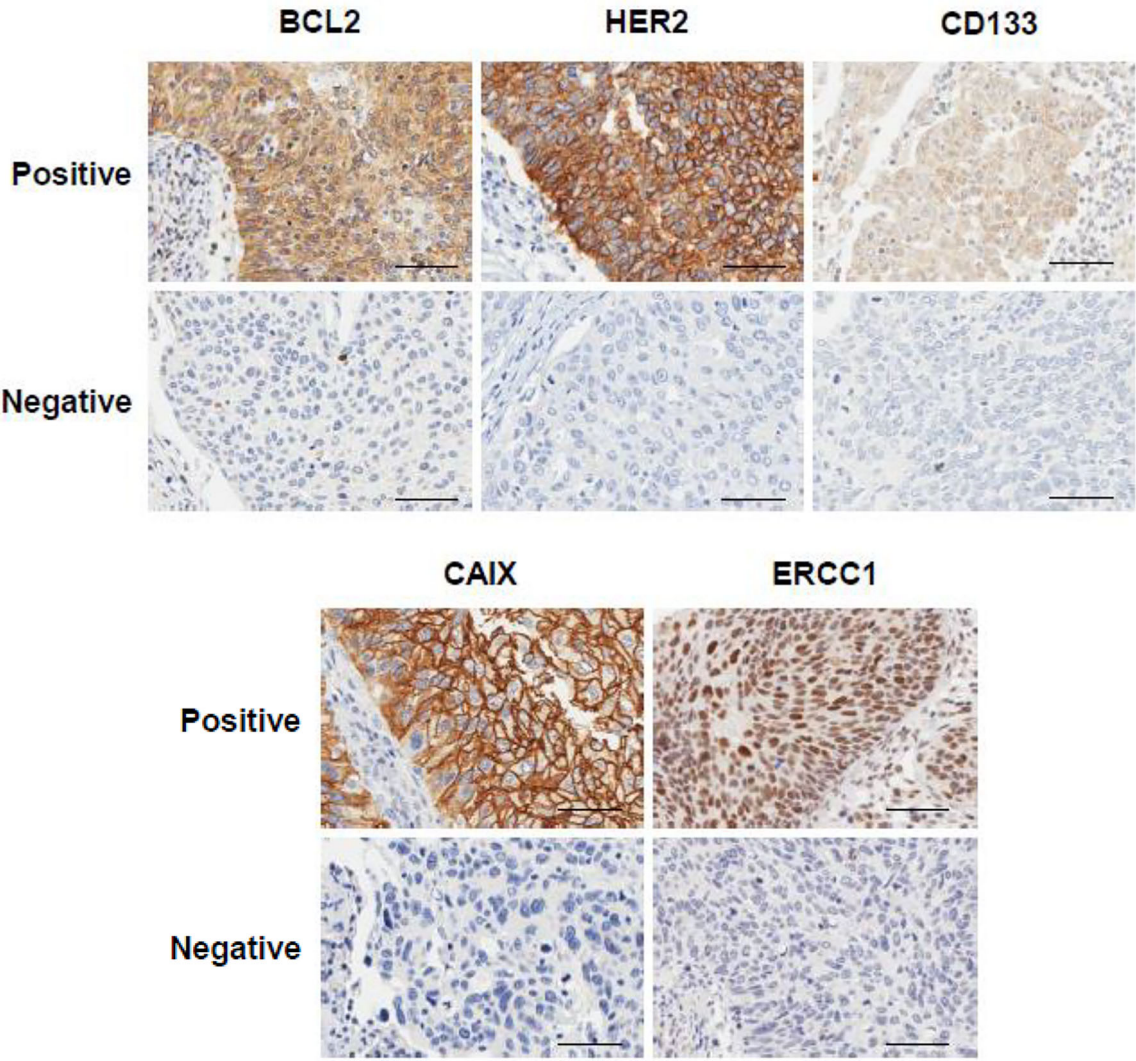
Expression of BCL-2, HER2, CD133, CAIX, and ERCC1 in early cervical cancer patients. Representative immunohistochemical images of positive and negative expression of each protein. The scale bar represents 50 μm.

To examine the expression level of the proteins, we assessed cervical cancer tissues using IHC. BCL-2 and CD133 proteins were observed mainly in the cytoplasm, HER2 and CAIX mainly in the cell membrane, and ERCC1 mainly in the nucleus. Representative IHC images of these proteins are shown in **Figure 1**. We used histoscore to compare the extent of overall protein expression. CAIX and ERCC1 were expressed more than the other three proteins in early cervical cancer tissues. To examine the mRNA expression of the five proteins, we analyzed the GEO database (GSE44001), which contains the results of a DASL assay for RNA profiling with paraffin tissue from 300 patients with cervical cancer. We examined the correlation between each protein and mRNA. CD133 and CAIX protein expression was correlated weakly with mRNA expression (r=0.155; p=0.021 and r=0.190; p=0.005). Although other proteins had no statistical significance, the correlation had a positive trend (**Supplementary Figure 1**).

CD133 and CAIX expression was cell type-dependent; CD133 and CAIX were more highly expressed in adeno/adenosquamous carcinoma (p=0.030 and p=0.003, respectively) (**Table 2**). These results suggest that CD133 and CAIX have different roles in cervical cancer according to cell type. In addition, the higher expression of ERCC1 was negatively correlated with the depth of invasion (p=0.013) and higher expression of CD133 was negatively correlated with high-risk type HPV infection (p=0.034). When protein expression level was analyzed in the adjuvant treatment group, CAIX positive was more frequent in the chemoradiosensitive group (58.9% *vs*. 35.0%, p=0.082), and ERCC1 positive was more frequent in the chemoradioresistant group (68.4% *vs*. 42.0%, p=0.059), though statistically not significant (**Table 3**). The two proteins should further be investigated as markers of chemoradiosensitivity in early cervical cancers.

**Table 2:** The correlation between expression of potential protein markers predicting chemoradioresistance with clinicopathologic. characteristics of early cervical cancer.

	No.	BCL-2		HER2		CD133		CAIX		ERCC1	
		Histoscore[95% CI]	*p* value	Histoscore[95% CI]	*p* value	Histoscore[95% CI]	*p* value	Histoscore[95% CI]	*p* value	Histoscore[95% CI]	*p* value
Stage			0.079		0.931		0.810		0.908		0.286
IB1/IIA	291	19 [14 - 24]		13 [10 - 17]		1 [1 - 1]		33 [26 – 41]		70 [62 – 78]	
IB2/IIB	45	10 [1- 19]		13 [6 - 20]		1 [0 – 2]		34 [26 – 41]		59 [39 - 78]	
Histology			0.078		0.398		0.030		0.003		0.505
SCC	256	19 [14 - 25]		12 [9 - 15]		1 [0 - 1]		26 [21 – 31]		67 [58 – 76]	
AD/ASC	80	11 [4 – 19]		17 [7 – 27]		1 [0 – 2]		59 [38 – 79]		73 [58 – 89]	
Tumor size			0.053		0.452		0.266		0.068		0.097
≤ 4cm	256	20 [14 - 25]		14 [10 - 18]		1 [1 - 1]		30 [23 – 37]		72 [63 – 81]	
> 4cm	80	11 [4 - 18]		11 [6 – 17]		1 [0 – 3]		44 [31 – 58]		58 [43 – 73]	
High-risk HPV infection			0.999		0.096		0.034		0.490		0.125
Negative	37	19 [4 - 33]		9 [3 - 14]		2 [1 - 3]		25 [7 - 43]		54 [30 - 78]	
Positive	128	19 [11 - 26]		16 [9 - 23]		0 [0 - 1]		32 [22 - 42]		75 [61 - 89]	
Depth of invasion			0.828		0.633		0.293		0.523		0.013
≤ 50%	108	17 [9 - 24]		15 [7 - 23]		1 [0 – 1]		30 [17 - 43]		85 [69 - 102]	
> 50%	228	18 [12 - 23]		13 [10 - 16]		1 [1 - 2]		35 [27 - 42]		62 [54 - 70]	
PM invasion			0.541		0.452		0.254		0.416		0.342
Negative	305	18 [13 - 23]		14 [10 - 17]		1 [1 - 1]		33 [26 - 39]		70 [62 - 78]	
Positive	31	14 [1 - 27]		10 [2 - 19]		1 [0 - 1]		42 [19 - 66]		57 [32 - 82]	
Resection margin			0.782		0.105		0.760		0.826		0.794
Negative	323	17 [13 - 22]		12 [9 - 16]		1 [1 - 1]		33 [27 - 40]		69 [61 - 77]	
Positive	13	22 [15 - 59]		35 [7 - 62]		1 [0 - 2]		37 [2 - 72]		64 [22 - 106]	
LN metastasis			0.389		0.524		0.498		0.372		0.328
Negative	256	19 [13 - 24]		14 [10 - 18]		1 [0 - 1]		32 [24 - 39]		71 [62 - 80]	
Positive	80	14 [6 - 22]		12 [7 - 17]		1 [0 - 2]		39 [25 - 53]		62 [46 - 77]	
Primary Treatment			0.170		0.427		0.649		0.615		0.055
No adj.	171	21 [14 - 28]		15 [9 - 20]		1 [1 - 1]		35 [24 - 46]		76 [65 - 88]	
Adj.	165	15 [9 - 20]		12 [8 – 16]		1 [0 - 2]		32 [24 - 39]		61 [51 - 72]	

SCC, squamous cell carcinoma; AD, adenocarcinoma; ASC, adenosquamous cell carcinoma; PM, parametrium; LN, lymph node; adj., adjuvant treatment.

All histoscore values are mean.

**Table 3:** Expression of protein markers according to chemoradioresistance in 133 early cervical cancer patients treated with operation and adjuvant treatment.

	Sensitive (*n *= 113)	Resistant (*n *= 20)	*p* value
BCL-2			0.421
Low expression	70 (62.5%)	10 (50.0%)	
High expression	42 (37.5%)	10 (50.0%)	
HER2			0.225
Low expression	59 (52.7%)	7 (35.0%)	
High expression	53 (47.3%)	13 (65.0%)	
CD133			0.587
Low expression	93 (83.0%)	15 (75.0%)	
High expression	19 (17.0%)	5 (25.0%)	
CAIX			0.082
Low expression	46 (41.1%)	13 (65.0%)	
High expression	66 (58.9%)	7 (35.0%)	
ERCC1			0.059
Low expression	65 (58.0%)	6 (31.6%)	
High expression	47 (42.0%)	13 (68.4%)	

### Prognostic Significance of BCL-2, HER2, CD133, CAIX, and ERCC1 Expressions in Early Cervical Cancer

**Figure 2:**
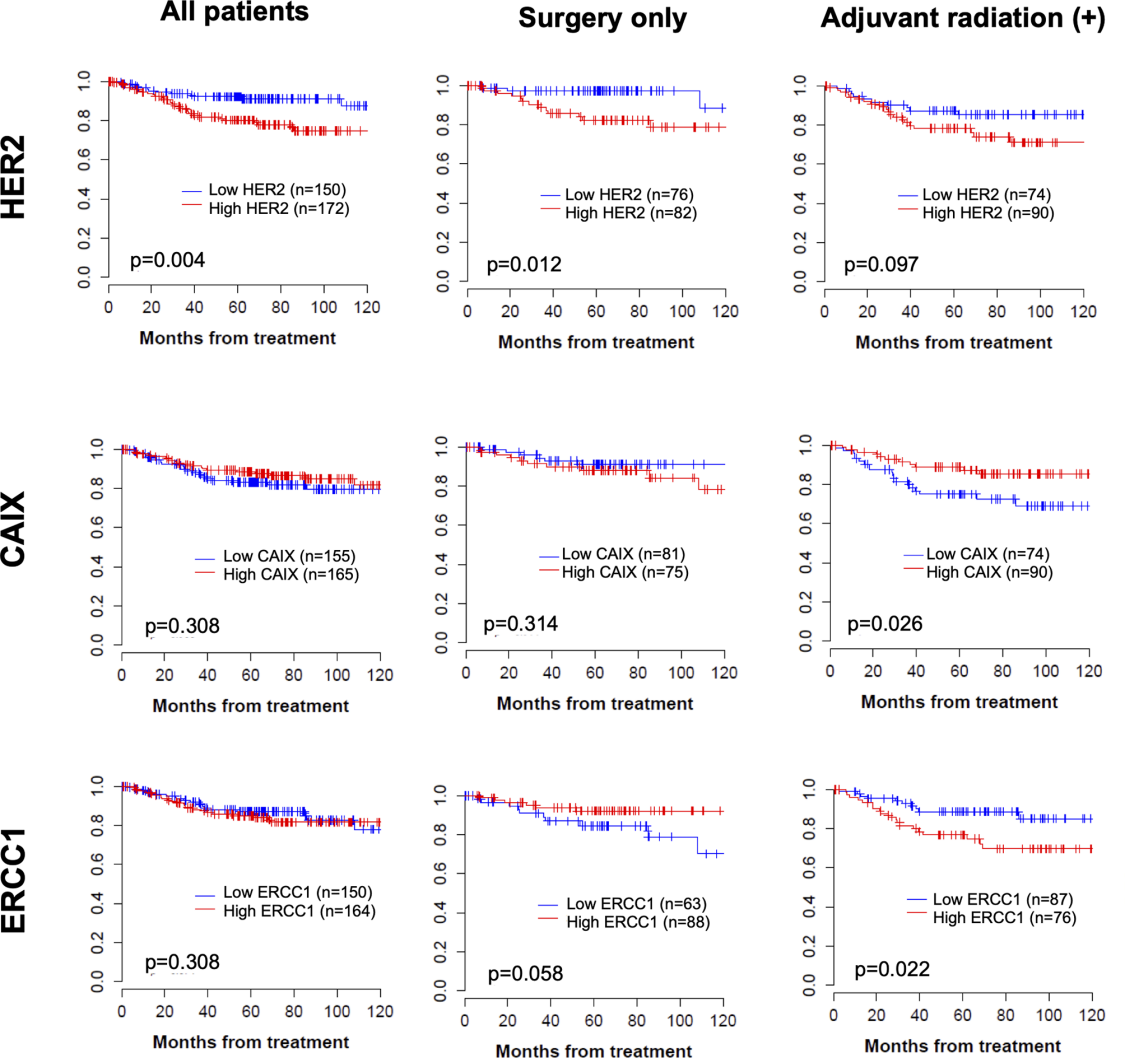
Kaplan–Meier curve of disease-free survival according to each protein expression in total group, operation only group, and operation followed by adjuvant treatment group.

In total, there was a median follow-up period of 66 months (range 1–143) and a 5-year DFS of 87% (95% CI, 83–91). Patients with higher HER2 expression had significantly poor DFS compared to the total group (80.1% *vs*. 92.2%, p=0.004; **Figure 2**). In subgroup analysis, high HER2 expression was also associated with poor DFS (p=0.012) and low ERCC1 expression tended to be associated with inferior DFS in the non-adjuvant treatment group (p=0.058; **Figure 2**). In the adjuvant radiation group, patients with higher expression of CAIX had significantly better DFS (89.0% *vs*. 75.0%, p=0.026, **Figure 2**). Interestingly, ERCC1 expression was associated with poorer DFS (76.1% *vs*. 88.9%, p=0.022) in the adjuvant radiation group, though it was a favorable marker in the surgery only group. In high-risk group, such as lymph node metastasis or large tumor size, the gap in DFS became wider with higher protein expression, especially HER2 and ERCC1 (**Supplementary Figure 2**). This indicated that the protein expression level could be a more unfavorable factor in cervical cancer patients with high-risk factors. The infection of HPV and its two viral oncoproteins, E6 and E7 that cause tumorigenic transformation of cervical epithelium was also analyzed. In **Supplementary Table 2**, it has been shown that high risk HPV infected patients are more likely to be radiosensitive. In Kaplan-Meier curve, HPV infection has a trend of better DFS in adjuvant radiation group, however, it was not statistically significant (see **Supplementary Figure 3**).

Using the Cox proportional analysis, the association between prognostic values and disease recurrence was analyzed in all the patients (**Table 4**). Clinicopathologic factors, histology, and lymph node metastasis were independent predictors of DFS (hazard ratio, HR=3.52, 95% CI 1.96–6.32, p<0.001; HR=3.69, 95% CI 1.95–6.99, p=0.001) in the multivariate analysis. High expression of HER2 was an independent prognostic factor for DFS (HR=2.10, 95% CI 1.08–4.07, p=0.029), which persisted as a prognostic marker in the non-adjuvant treatment group. In the adjuvant radiation group, low expression of CAIX was an independent prognostic value for DFS (HR=0.45, 95% CI 0.21–0.95, p=0.037).

**Table 4:** Multivariate analysis of the association between prognostic variables and disease-free survival in cervical cancer patients according to adjuvant treatment.

	Total group		Operation and adjuvant treatment group	
	HR (95% CI)	*p* value	HR (95% CI)	*p* value
Stage (IB2/IIB)	1.67 [0.83 - 3.36]	0.150	2.06 [0.94 - 4.52]	0.070
Histology (AD/ASC *vs* SCC)	3.52 [1.96 - 6.32]	<0.001	5.12 [2.47 - 10.62]	<0.001
Tumor size (> 4 cm)	1.04 [0.86 – 1.24]	0.712	0.89 [0.68 – 1.16]	0.399
Parametrial involvement	1.33 [0.57 - 3.07]	0.507	1.35 [0.51 – 3.55]	0.540
Lymph node metastasis	3.69 [1.95 - 6.99]	<0.001	2.51 [1.14 - 5.50]	0.022
BCL-2 (+)	1.22 [0.67 - 2.22]	0.516	1.67 [0.79 - 3.56]	0.183
HER2 (+)	2.10 [1.08 - 4.07]	0.029	1.58 [0.72 - 3.46]	0.255
CD133 (+)	0.96 [0.47 - 1.96]	0.914	0.56 [0.21 - 1.49]	0.248
CAIX (+)	0.68 [0.37 - 1.23]	0.200	0.45 [0.21 - 0.95]	0.037
ERCC1 (+)	1.03 [0.55 - 1.91]	0.937	1.57 [0.69 - 3.54]	0.279

SCC, squamous cell carcinoma; AD, adenocarcinoma; ASC, adenosquamous cell carcinoma.

With data of previous article, we compared the results of univariate analysis whether each of the markers are independently significant and have similar effect in both early and advanced stage (**Supplementary Table 3**). High expression of HER2 was an independent prognostic factor of disease recurrence and overall survival in early cervical cancer. However, the significance of HER2 in disease recurrence had decreased in locally advanced cervical cancer. Also, of early cervical cancer patients, low expression of CAIX was an independent prognostic value for DFS in adjuvant radiation group. Patients with locally advanced cervical cancer had almost received adjuvant radiation, and low CAIX expression tended to be associated with disease recurrence (HR=0.73, 95% CI 0.51–1.04, p=0.078).

### Assessment of the Prognostic Power of the Combined Clinical–Molecular Model

**Figure 3:**
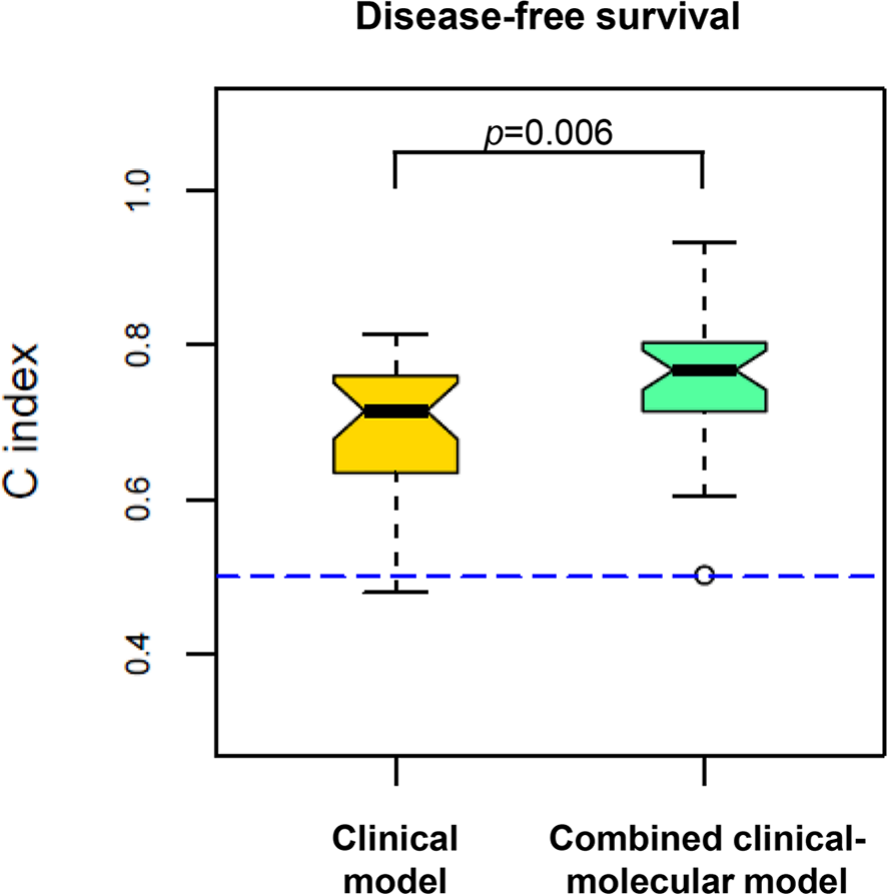
Comparison of predictive power in disease recurrence between the clinical model (yellow, left) and the combined clinical–molecular model (green, right). The plots show the distribution of 100 C-indexes and are compared by using the Wilcoxon signed-rank test. The combined clinical–molecular model had better performance in predicting disease recurrence (median C-index 0.77) compared to the clinical model (median C-index 0.71; p=0.006). The dashed line indicates the C-index equivalent to a random guess (C-index=0.50).

To examine whether the data associated with the five proteins enhanced the prognostic power of the clinical data, we compared the C-index between the clinical model and the combined clinical–molecular model to predict disease recurrence. Importantly, the combined clinical–molecular model predicted recurrence (mean C-index, 0.77; range, 0.50–0.93) with significantly improved power compared to the clinical model (mean C-index, 0.71; range, 0.48–0.81) (p=0.006, **Figure 3**).

## Discussion

In the present study, we investigated the prognostic significance of BCL-2, HER2, CD133, CAIX, and ERCC1 expression in early-stage cervical cancer because they were prognostic factors in locally advanced cervical cancer. We identified that each protein had a different implication in early cervical cancer. There was a trend of low expression of CAIX and high expression of ERCC1 in patients with a resistant response to adjuvant treatment. Furthermore, high HER2 expression predicted an unfavorable oncologic outcome, and low CAIX and high ERCC1 expression predicted an unfavorable response to adjuvant treatment in patients with early cervical cancer. In addition, we demonstrate for the first time that ERCC1 had a different association with DFS depending on whether patients received adjuvant treatment; low expression with poor DFS in the group that received no adjuvant and high expression with poor DFS in the adjuvant radiation group. Based on these results, we suggest that this is the first study to validate the prognostic significance of proteins, which are important in locally advanced cancer, in a cohort with early cervical cancer. HER2, CAIX, and ERCC1 may be useful as predictive markers in chemoradioresistance and prognostic markers in the recurrence of cervical cancer.

Carbonic anhydrase IX (CAIX) is a transmembrane protein that catalyzes the reversible hydration of carbon dioxide to carbonic acid, regulating intracellular pH and maintaining a normal pH in tumor cells under hypoxic conditions (20). Therefore, it is a useful endogenous marker of tumor hypoxia and a predictor of radiation-resistant hypoxic cells. Other studies refute the relationship between CAIX expression and hypoxia (21, 22). Similarly, the prognostic significance of CAIX is controversial. Some studies have found significant associations between CAIX and poor prognosis in locally advanced cervical cancer (23, 24). However, other studies have shown no significant association (21, 25) or that the high expression of CAIX is related to better survival (26). Our study showed that CAIX expression is associated with RT response and that the low expression of CAIX is related with a poor response to radiation in early cervical cancer patients. The discrepancy between CAIX expression and RT susceptibility in the current study suggests that other factors may be associated with hypoxia, rather than CAIX expression.

In the locally advanced stage or early stage of cervical cancer with risk factors, patients receive radiotherapy or CCRT with cisplatin. The main cytotoxic activity of cisplatin is based on the formation of DNA adducts, which trigger a series of intracellular events that ultimately result in cancer cell death (27, 28). Excision repair cross-complementing 1 (ERCC1) is a key protein in the nucleotide excision repair (NER) pathway, which recognizes and removes cisplatin-induced DNA adducts, decreasing the cell response to cisplatin (29). In our study, high ERCC1 expression was associated with poor DFS in patients with adjuvant radiotherapy or CCRT with cisplatin. This is because higher ERCC1 expression increases the repair of DNA adducts induced by cisplatin, which results in a poor response to treatment and unfavorable oncologic outcomes. A significant correlation between ERCC1 mRNA expression levels and cisplatin resistance has been demonstrated in cervical cancer cell lines (30) and several studies in patients with locally advanced cervical cancer have arrived at similar results (31, 32). In normal cells, impaired DNA repair may lead to cell toxicity or genomic instability, a critical step in cancer pathogenesis. Therefore, the levels of ERCC1 were significantly lower in cancer patients than in normal controls (33). In addition, the International Adjuvant Lung Cancer Trial (IALT) showed that those with ERCC1-positive tumors survived longer than those with ERCC1-negative tumors among patients who did not receive platinum-based chemotherapy (34). This can explain why the low expression of ERCC1 is associated with poor DFS in patients who were only treated with surgery in our study.

HER2 is one of the EGFR family and its expression in cervical cancer ranges from 1 to 12%. Several studies have found that HER2 expression is an independent predictor of poor prognosis in cervical cancer (13, 35). Our study also showed that high HER2 expression was significantly associated with poor survival in patients with early cervical cancer. However, other studies have revealed that there was no association with unfavorable outcome (36, 37).

CD133 is probably one of the most studied markers in cancer stem cells. High expression of CD133 expression has been correlated with poor prognostic features and chemoresistance (38). In the locally advanced stage of cervical cancer, patients expressing a high level of CD133 demonstrated a better response to CCRT (14). BCL-2 is an anti-apoptotic molecule and the expression of BCL-2 and BAX might correspond to disease stage progression or cell radiosensitivity in cervical cancer (39). However, there was no association with the expression of CD133 or BCL-2 and disease recurrence or chemoradioresistance in this study.

RPPA can identify proteomic profiling of clinical samples and the quantitative detection of signaling proteins by detecting three-dimensional epitope structure in fresh frozen samples (40). This is a powerful approach for identifying and validating targets, classifying tumor subsets, assessing pharmacodynamics, and identifying prognostic and predictive markers, adaptive responses, and rational drug combinations in model systems and patient samples (41). However, the long-term preservation of high-quality specimens such as frozen tissue is not practical in a routine clinical care environment. The tissues of patients are usually FFPE because this is the most common tissue preparation method for diagnostic histopathology and can be stored for archival purposes. IHC is well-established and commonly used in histopathology for diagnosis, prognosis, and biomarker identification. In this context, we examined the possibility that potential protein markers predict chemoradioresistance in early cervical cancer. We identified each protein expression by IHC and the results was that BCL-2 and CD133 were stained in mainly cytoplasm, HER2 and CAIX in cell membrane, and ERCC1 in nucleus and the expression level was measured with ‘histoscore’ in various range. Further studies using fresh frozen and FFPE paired tissues using both IHC and RPPA are needed to translate these findings into clinical applications.

There are a few limitations in this study. First, we used conventional IHC methods for the quantification of these 5 markers. Despite its increasing role in the clinic, IHC still presents with challenges such as inter-assay variability of antibody clones, intra-tumor heterogeneity, and lack of optimal scoring systems and standard cut-off values for positivity. To account for such variability, commercially available clones of all 5 markers, which were previously validated in locally advanced cervical cancer specimens (**Supplementary Figure 4**), were used under the supervision of experienced pathologists, and the Cox model of disease-free survival using R software was adopted to determine the optimal cut-off values for each marker. Further studies are needed to develop standardization for clinical utility. Second, these proteins were not identified continuously, and the expression level was almost negative, especially in CD133, making it difficult to apply the predictive model which was identified in Choi et al. (14). Third, the study design was retrospective and included a relatively small population treated with adjuvant radiotherapy at a single institution. Further studies with prospective design and a larger multicenter cohort are necessary to validate the association of the factors and chemoradioresistance.

HPV is the most common cause of cervical cancer. Among them, the E6 and E7 oncoproteins are thought to be mainly responsible for malignant conversion by inducing disruptions in transmembrane signaling, regulation of the cell cycle, which consequently result in the transformation of established cell lines, immortalization of primary cell lines, and disregulation of chromosomal stability. These interactions occur with the inactivation of tumor suppressors p53 and/or pRB (42, 43). Studies of proteomics related to HPV oncoproteins have been continued to identify potential therapeutic approaches in cervical cancer and some proteins in phosphoinositide 3-kinase (PI3K)/protein kinase B (Akt) pathways have been found to be closely related with HPV oncoproteins (44, 45). The results of the present study along with those from our previous ones (14), BCL2, HER-2, CD133, CAIX and ERCC1 were revealed to have significant associations with cervical cancer prognosis. These findings warrant future studies to further identify any potential influences of HPV oncoproteins in disease development.

In conclusion, the present study used immunohistochemical staining to validate how the expression of BCL-2, HER2, CD133, CAIX, and ERCC1 could predict chemoradioresistance and disease recurrence in patients with early cervical cancer. CAIX and ERCC1 showed a trend in expression level according to the response to adjuvant radiotherapy or chemoradiotherapy. A lower expression of CAIX and overexpression of ERCC1 were independently poor prognostic factors of recurrence in patients with adjuvant treatment. Overexpression of HER2 was also associated with unfavorable disease prognosis in early cervical cancer. Each protein had a different association with disease recurrence in early-stage cervical cancer and this result was not similar to that found in locally advanced cervical cancer. This information could improve our understanding of the necessity of applying predictive factors adequately according to patient clinical factors.

## Data Availability Statement

The raw data supporting the conclusions of this article will be made available by the authors, without undue reservation.

## Ethics Statement

The studies involving human participants were reviewed and approved by the Institutional Review Board of Samsung Medical Center (IRB no. SMC 2009-09-002 and 2015-07-122; Seoul, South of Korea). The patients/participants provided their written informed consent to participate in this study.

## Author Contributions

SJ: Conceptualization, Formal analysis, Data curation, Writing- original draft. J-YC: Methodology, Investigation, Writing- review & editing. S-JB: Methodology, Investigation. CK, Y-YL, T-JK, J-WL, B-GK, YC, and SO: Resources. CC: Formal analysis, Investigation, Resources, Data curation, Writing-review & editing, Supervision. All authors contributed to the article and approved the submitted version.

## Funding

This research was supported by funding from the National Research Foundation of Korea (NRF-2017R1D1A1B05035844).

## Conflict of Interest

The authors declare that the research was conducted in the absence of any commercial or financial relationships that could be construed as a potential conflict of interest.

## Supplementary Material

Supplementary Figure 1 Correlations between each protein and messenger RNA (mRNA) expression. mRNA expression level was measured by microarray gene expression profiling, whereas the protein expression was assessed by immunohistochemistry. CD133 and CAIX protein expression was correlated with mRNA expression (Spearman’s rho (r)=0.155; p=0.021, and r=0.190; p=0.005, respectively). Red color represent recurrence, and triangular shape represent adjuvant radiation.

Supplementary Figure 2 Kaplan-Meier curve of disease-free survival according to each protein expression by status of **(A)** lymph node metastasis and **(B)** tumor size.

Supplementary Figure 3 Kaplan-Meier curve of disease-free survival according to status of **(A)** HPV infection **(B)** high risk HPV infection in total, operation, and operation and adjuvant treatment group.

Supplementary Figure 4
**(A)** Western blot data from locally advanced cervical cancer patient specimens **(B)** Correlations between IHC score and Western blotting of each proteins.
