# Supplementary figures and images for: Validation of Potential Protein Markers Predicting Chemoradioresistance in Early Cervical Cancer by Immunohistochemistry
Source: Front Oncol. 2021 Jul 19;11:665595. doi: 10.3389/fonc.2021.665595 (PMC8327183; doi:10.3389/fonc.2021.665595)

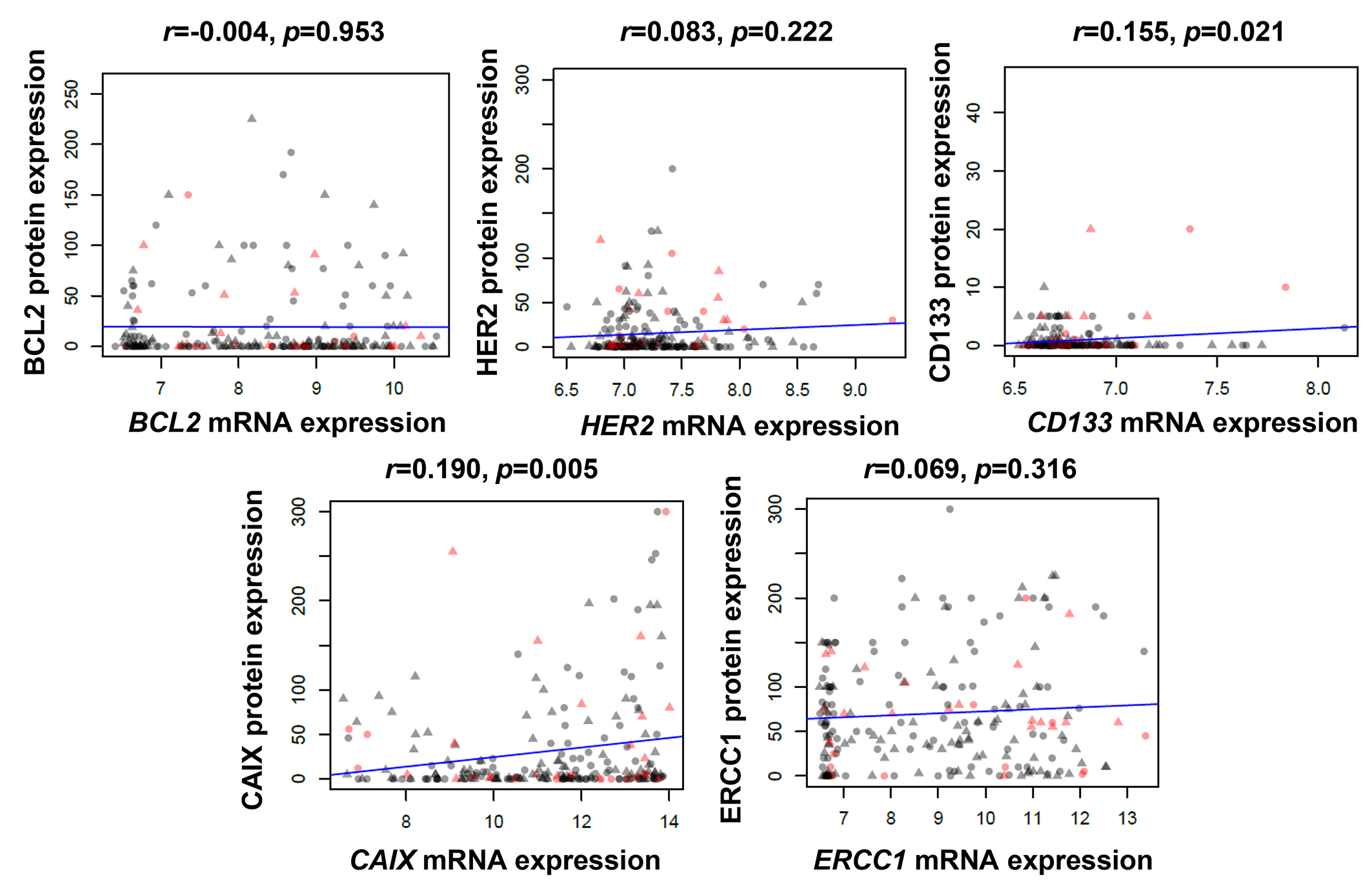

Supplement: Supplementary Figure 1 — Correlations between each protein and messenger RNA (mRNA) expression. mRNA expression level was measured by microarray gene expression profiling, whereas the protein expression was assessed by immunohistochemistry. CD133 and CAIX protein expression was correlated with mRNA expression (Spearman’s rho (r)=0.155; p=0.021, and r=0.190; p=0.005, respectively). Red color represent recurrence, and triangular shape represent adjuvant radiation. [file Image_1.tif]

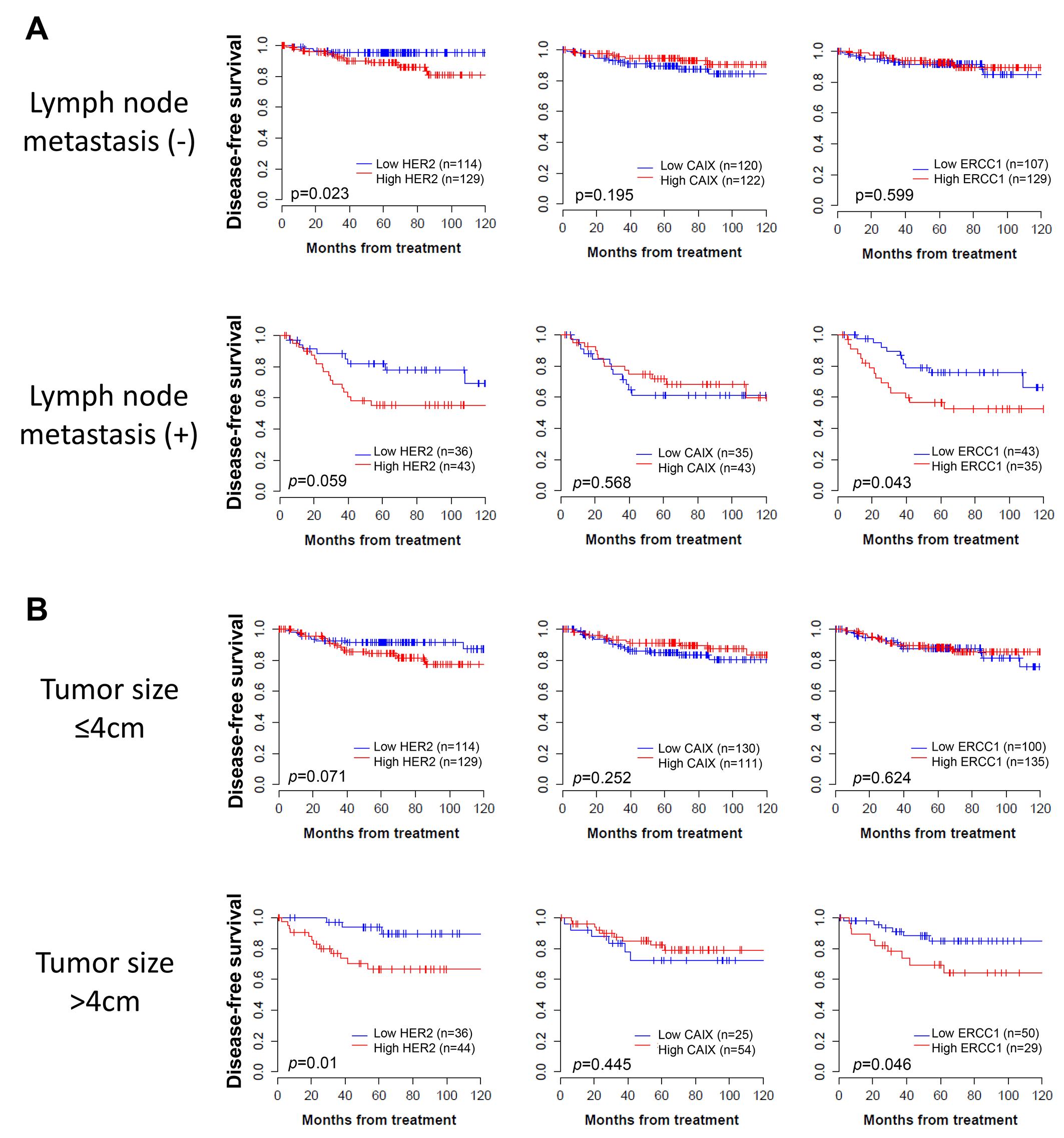

Supplement: Supplementary Figure 2 — Kaplan-Meier curve of disease-free survival according to each protein expression by status of (A) lymph node metastasis and (B) tumor size. [file Image_2.tif]

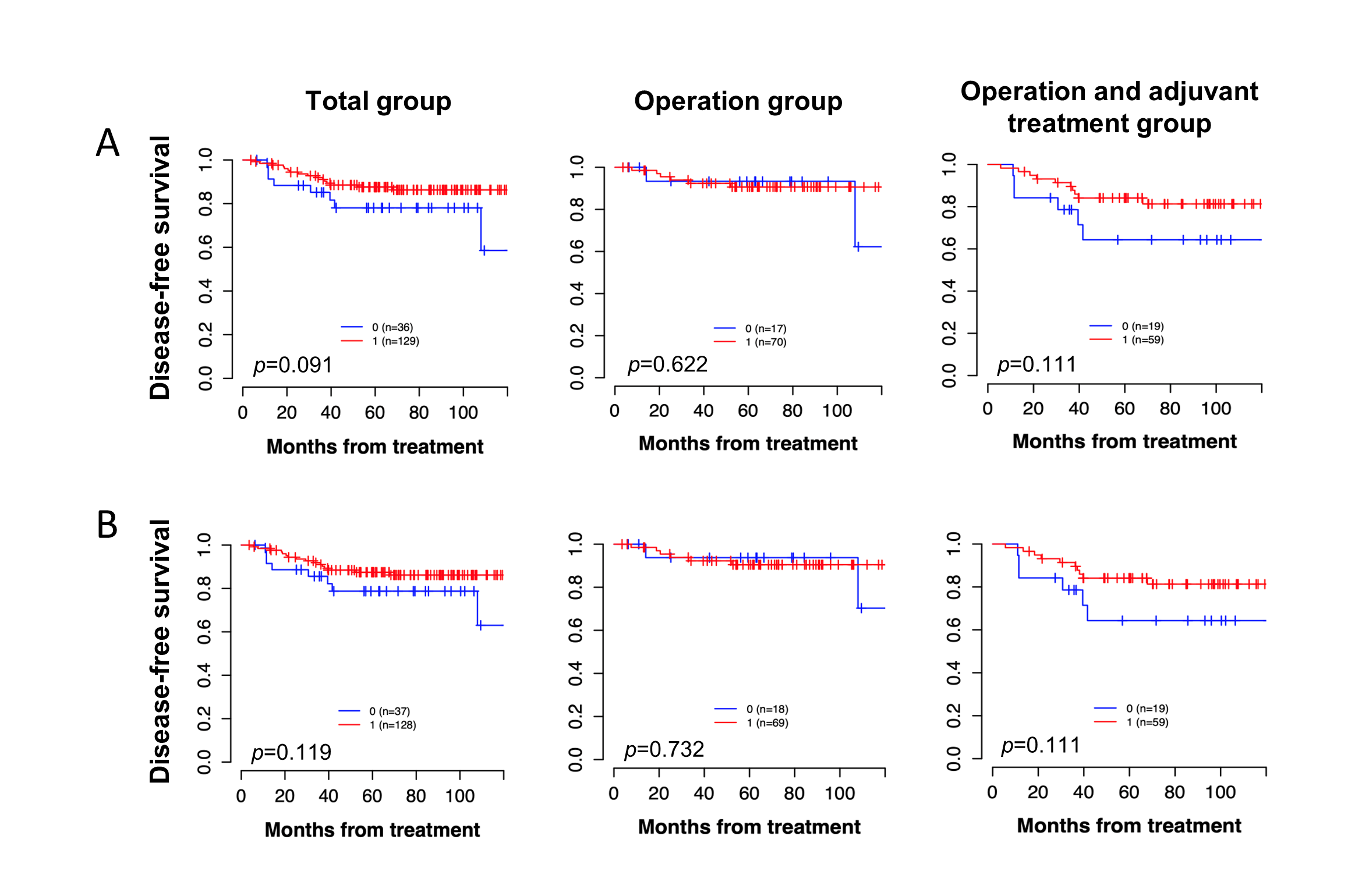

Supplement: Supplementary Figure 3 — Kaplan-Meier curve of disease-free survival according to status of (A) HPV infection (B) high risk HPV infection in total, operation, and operation and adjuvant treatment group. [file Image_3.tif]

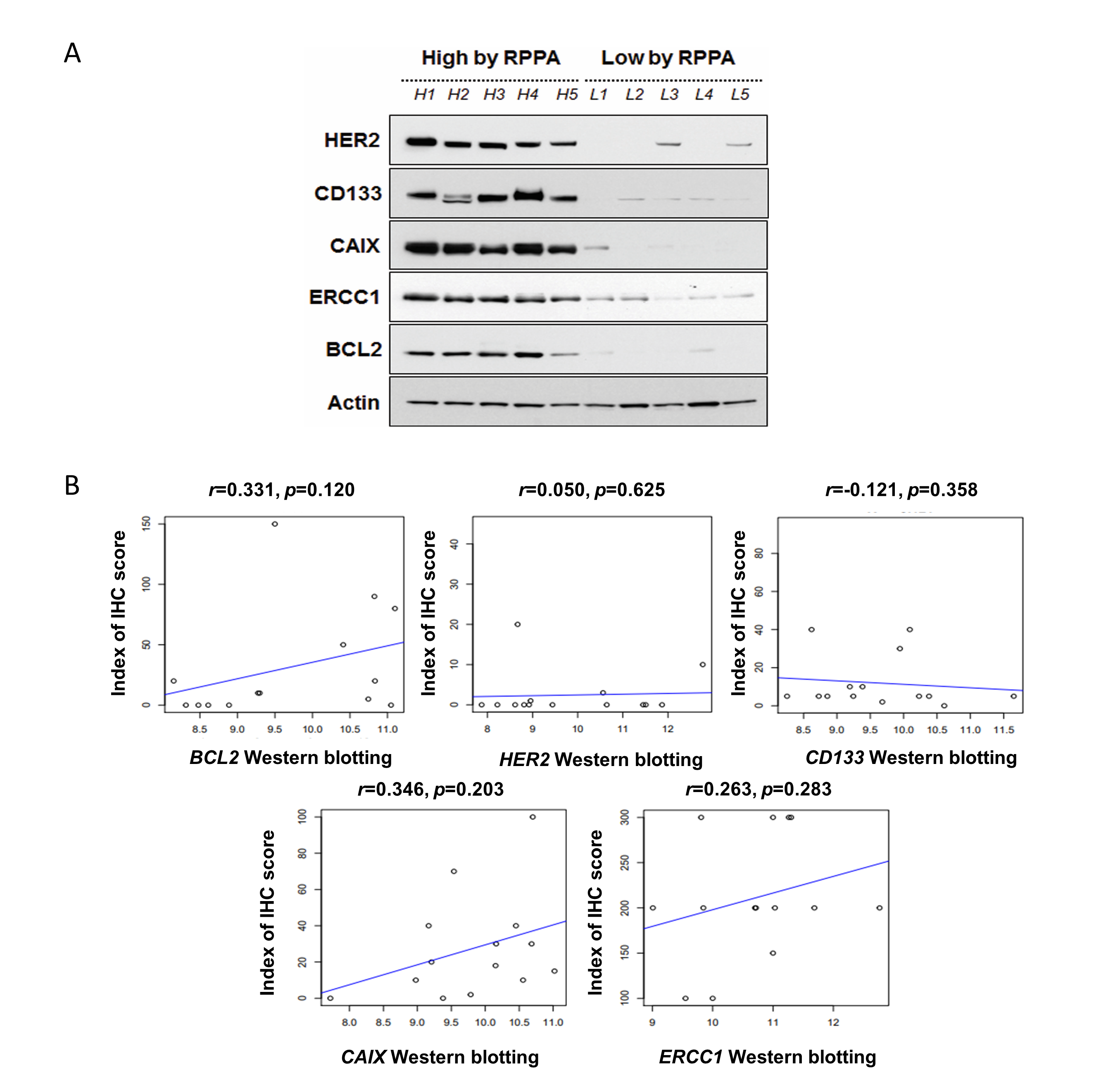

Supplement: Supplementary Figure 4 — (A) Western blot data from locally advanced cervical cancer patient specimens (B) Correlations between IHC score and Western blotting of each proteins. [file Image_4.tif]
