# Supplementary material for: Validation of Potential Protein Markers Predicting Chemoradioresistance in Early Cervical Cancer by Immunohistochemistry
Source: Front Oncol. 2021 Jul 19;11:665595. doi: 10.3389/fonc.2021.665595 (PMC8327183; doi:10.3389/fonc.2021.665595)
Supplement: Supplementary file 5 [file Table_1.docx]

**Supplementary Table 1.** Antibodies used in immunohistochemistry

| Antibody | Vendor | Clonality | Cat. # | Incubation at RT | Dilutions | Antigen retrieval | Positive control |
| --- | --- | --- | --- | --- | --- | --- | --- |
| BCL-2 | Dako | Mono mouse (Clone 124) | M0887 | 15 min | 1:100 | 20 min, ER1 buffer  (pH 6.0) | Tonsil tissues |
| HER2 | Dako | Poly rabbit | K5207 | 30 min | Ready to use | 40 min, steamer  (pH 6.0) | Tonsil tissues |
| CD133 | Fitzgerald | Poly rabbit | 70R-13813 | 40 min | 1:200 | 20 min, steamer,  LOW buffer  (pH 6.0) | Kidney tissues |
| CAIX | Abcam | Poly rabbit | Ab15086 | 15 min | 1:2000 | 20 min, ER1 buffer  (pH 6.0) | Kidney tissues |
| ERCC1 | GeneTex | Mono mouse (Clone 8F1) | GTX22356 | 15 min | 1:150 | 20 min, steamer,  ER2 buffer  (pH 8.0) | Tonsil tissues |

RT, Room temperature
