# Supplementary material for: Validation of Potential Protein Markers Predicting Chemoradioresistance in Early Cervical Cancer by Immunohistochemistry
Source: Front Oncol. 2021 Jul 19;11:665595. doi: 10.3389/fonc.2021.665595 (PMC8327183; doi:10.3389/fonc.2021.665595)
Supplement: Supplementary file 6 [file Table_2.docx]

**Supplementary Table 2.** Clinicopathologic characteristics according to chemoradioresistance

|  | Sensitive  (*n*=113) | Resistant  (*n*=20) | *p* value |
| --- | --- | --- | --- |
| Stage |  |  | 0.141 |
| IB1/ IIA | 93 (82.3%) | 13 (65.0%) |  |
| IB2/ IIB | 20 (17.7%) | 7 (35.0%) |  |
| Histology |  |  | 0.001 |
| SCC | 94 (83.2%) | 9 (45.0%) |  |
| AD/ASC | 19 (16.8%) | 11 (55.0%) |  |
| Tumor size |  |  | 1.000 |
| ≤ 4 cm | 72 (63.7%) | 13 (65.0%) |  |
| > 4 cm | 41 (36.3%) | 7 (35.0%) |  |
| Adjuvant treatment |  |  | 0.132 |
| RT | 52 (46.0%) | 5 (25.0%) |  |
| CCRT | 61 (54.0%) | 15 (75.0%) |  |
| High risk HPV infection |  |  | 0.173 |
| Negative | 10 (17.9%) | 4 (44.4%) |  |
| Positive | 46 (82.1%) | 5 (55.6%) |  |

SCC, squamous cell carcinoma; AD, adenocarcinoma; ASC, adenosquamous cell carcinoma; RT, radiotherapy; CCRT, concurrent chemoradiotherapy
