# Supplementary material for: Validation of Potential Protein Markers Predicting Chemoradioresistance in Early Cervical Cancer by Immunohistochemistry
Source: Front Oncol. 2021 Jul 19;11:665595. doi: 10.3389/fonc.2021.665595 (PMC8327183; doi:10.3389/fonc.2021.665595)
Supplement: Supplementary file 7 [file Table_3.docx]

**Supplementary table 3.** Comparison the association between prognostic variables and survivals in early stage and locally advanced stage cervical cancer patients according to univariate analysis

|  | Early stage | | Locally advanced stage | |
| --- | --- | --- | --- | --- |
|  | HR (95% CI) | *p* value | HR (95% CI) | *p* value |
| Overall survival |  |  |  |  |
| BCL-2 (+) | 2.08 [0.86 – 5.04] | 0.105 | 1.33 [0.92 – 1.93] | 0.126 |
| HER2 (+) | 2.84 [1.03 – 7.83] | 0.043 | 1.37 [1.01 – 1.84] | 0.041 |
| CD133 (+) | 1.25 [0.45 – 3.44] | 0.669 | 1.01 [0.67 – 1.53] | 0.956 |
| CAIX (+) | 0.86 [0.36 – 2.07] | 0.734 | 0.74 [0.50 – 1.11] | 0.144 |
| ERCC1 (+) | 1.34 [0.55 – 3.27] | 0.525 | 1.35 [0.88 – 2.05] | 0.167 |
| Disease-free survival |  |  |  |  |
| BCL-2 (+) | 1.13 [0.63 - 2.02] | 0.694 | 1.06 [0.77 – 1.45] | 0.734 |
| HER2 (+) | 2.49 [1.31 - 4.74] | 0.005 | 1.15 [0.88 – 1.49] | 0.306 |
| CD133 (+) | 1.54 [0.82 - 2.89] | 0.178 | 0.90 [0.63 – 1.28] | 0.549 |
| CAIX (+) | 0.74 [0.41 - 1.32] | 0.310 | 0.73 [0.51 – 1.04] | 0.078 |
| ERCC1 (+) | 1.13 [0.63 - 2.04] | 0.674 | 1.13 [0.79 – 1.62] | 0.505 |
